# Pilot study of the Greek Interventional Geriatric Initiative to Prevent Cognitive Impairment and Disability in individuals with subjective cognitive decline: paving the way towards brain health clinics in Greece

**DOI:** 10.3389/fpsyt.2025.1514227

**Published:** 2025-03-18

**Authors:** Panagiotis Alexopoulos, Panagiotis Felemegkas, Xanthi Arampatzi, Evdokia Billis, Eleni Dimakopoulou, Polychronis Economou, George A. Dimakopoulos, Themis P. Exarchos, Maria Frounta, Parthenia Giannakopoulou, Kalliopi Kalaitzi, Maria - Lamprini Koula, Eftyhia Nastou, Maria Skondra, Paraskevi Sakka, Faidra Kalligerou, Nikolaos Skarmeas, Marianna Tsatali, Magdalini Krommyda, Maria Karala, Nikolaos Mastoras, Panagiotis Vlamos, Mary Yannakoulia, Ioannis Zaganas, Maria Karataraki, Maria Basta, Constantine Lyketsos

**Affiliations:** ^1^ Mental Health Services, Patras University General Hospital, Department of Medicine, School of Health Sciences, University of Patras, Patras, Greece; ^2^ Global Brain Health Institute, Medical School, Trinity College Dublin, The University of Dublin, Dublin, Ireland; ^3^ Department of Psychiatry and Psychotherapy, Klinikum rechts der Isar, Faculty of Medicine, Technical University of Munich, Munich, Germany; ^4^ Patras Dementia Day Care Centre, Corporation for Succor and Care of Elderly and Disabled-FRODIZO, Patras, Greece; ^5^ Alzheimer Athens, Athens, Greece; ^6^ Department of Physiotherapy, School of Health Rehabilitation Sciences, University of Patras, Patras, Greece; ^7^ Department of Civil Engineering (Statistics), School of Engineering, University of Patras, Patras, Greece; ^8^ Department of Informatics, Ionian University, Corfu, Greece; ^9^ Ageing Epidemiology Research Unit (AGE), School of Public Health, Imperial College London, London, United Kingdom; ^10^ The Longevity and Wellbeing Clinic, Athens, Greece; ^11^ Day Care Center for People with Dementia, Society of Psychosocial Research and Intervention, Ioannina, Greece; ^12^ First Department of Neurology, Eginition Hospital, School of Medicine, National and Kapodistrian University of Athens, Athens, Greece; ^13^ Department of Neurology, Columbia University, New York, NY, United States; ^14^ Network Aging Research, Heidelberg University, Heidelberg, Germany; ^15^ Department of Psychology, School of Humanities and Social Sciences, University of Western Macedonia, Kozani, Greece; ^16^ Laboratory of Psychology, Department of Cognition, Brain and Behavior, School of Psychology, Aristotle University of Thessaloniki (AUTh), Thessaloniki, Greece; ^17^ Department of Neurology, Queen’s Hospital, Romford, United Kingdom; ^18^ Department of Nutrition and Dietetics, Harokopio University, Athens, Greece; ^19^ Department of Neurology, Medical School, University of Crete, Heraklion, Greece; ^20^ Division of Psychiatry and Behavioral Sciences, School of Medicine, University of Crete, Heraklion, Greece; ^21^ Day Care Center for Alzheimer’s Disease PAGNH “Nefeli”, University Hospital of Heraklion, Heraklion, Greece; ^22^ Department of Psychiatry, University Hospital of Heraklion, Heraklion, Greece; ^23^ Richman Family Precision Medicine Center of Excellence, Department of Psychiatry and Behavioral Sciences at Johns Hopkins Bayview, Johns Hopkins School of Medicine, Baltimore, MD, United States

**Keywords:** dementia prevention, multi-dimensional, cognitive training, mental health, sensory loss, nutrition, cognitive behavioral therapy

## Abstract

The pilot phase of the Greek Interventional Geriatric Initiative to Prevent Cognitive Impairment and Disability (GINGER) aims to assess the feasibility of a multi-level dementia risk reduction intervention in individuals with subjective cognitive decline (SCD) over a six-month period. The study design incorporates a comprehensive set of trans-disciplinary assessments and interventions in multiple centers across Greece. Individuals 55 years or older with subjective cognitive complaints who do not fulfill criteria for either mild cognitive impairment or dementia are screened for dementia risk factors in the following domains: nutrition, physical activities, vision and hearing, vascular and metabolic parameters, anxiety and depressive symptoms, and insomnia. All GINGER participants receive a cognitive empowerment intervention. Using a precision medicine approach, they receive up to three additional domain-specific interventions based on their individual risk factor profiles. Changes in cognition, dementia risk factors, quality of life and other measures compared to baseline are assessed at three- and six months after the initiation of the intervention. The GINGER protocol was designed and is run by a multi-disciplinary team of dieticians, neurologists, psychiatrists, psychologists, and physiotherapists, while computer scientists oversee data management. The objectives of this pilot phase are (i) evaluation of the protocol’s feasibility, (ii) assessment of intervention effects on the individual risk domains targeted by the interventions, (iii) estimation of the overall effects of the intervention on cognitive function, dementia risk and quality of life. The GINGER findings will provide a solid foundation for paving the way towards a network of evidence-based brain health clinics in Greece.

## Introduction

Dementia represents a major challenge for public health today and is expected to strain healthcare systems globally in the coming decades. The term “dementia” refers to significant cognitive decline often caused by brain degenerative processes and/or vascular changes, rendering individuals unable to function independently in activities of daily living (1, 2). It is estimated that the number of people with dementia worldwide reached approximately 57 million in 2019 and is projected to surge to over 152 million by 2050, posing an unprecedented challenge to healthcare systems (3). In Greece, the number of people with dementia is expected to increase from around 206,000 in 2019 to approximately 300,000 by 2050, representing an increase of about 45% (3, 4). This underscores the importance of strategies and efforts to prevent or delay the onset of dementia.

Cognitive decline and the development of dementia is influenced by both non-modifiable and modifiable factors. Non-modifiable factors include advanced age and the presence of specific genes, which cannot be affected by available prevention strategies (5). In contrast, 45% of dementia cases worldwide can be attributed to modifiable risk factors, such as low education, hypertension, obesity, diabetes, smoking, alcohol consumption, sedentary lifestyle, depression, sensory loss, reduced social contacts, hearing loss, traumatic brain injuries, and air pollution (5). Interestingly, anxiety-, depression- and insomnia symptoms, as well as living alone are associated with the presence of subjective cognitive decline, which represents an at-risk phase, ideal for early intervention (6–8). All these factors indicate areas where intervention strategies could contribute to optimizing brain health, dementia risk reduction and improvement of quality of life (9). The World Health Organization released the first guidelines for reducing risk for cognitive decline and dementia in 2019 (10). These guidelines point to the usefulness of interventions targeting physical activity, social activity, cognitive empowerment, tobacco cessation, healthy dietary habits, alcohol reduction, as well as management of weight, diabetes, dyslipidemia, depression and sensory loss. In particular, age-related hearing loss might result in cognitive decline through reduced cognitive stimulation, loneliness, depression, social isolation, reduced cognitive reserve from decreased environmental stimuli, increased cognitive resources needed for listening and brain vascular changes (5). In addition, untreated visual loss embodies a risk factor for cognitive decline through the effects of diabetes-linked structural and functional brain changes, reduced cognitive stimulation, and/or shared neuropathological processes in both the retina and the brain (5).

In various countries, studies are being conducted and brain clinics have been established to reduce the risk of dementia through interventions targeting modifiable risk factors. For most of these initiatives the Finnish Geriatric Intervention Study to Prevent Cognitive Impairment and Disability (FINGER) serves as a model (11, 12). FINGER, a randomized controlled trial, demonstrated the feasibility and effectiveness of a two-year intervention in lifestyle modifications, including diet, physical exercise, cognitive training, and improvement of vascular and metabolic parameters. The interventions were delivered through both individual and group sessions. Individuals aged 60 to 77 with an increased risk of dementia participated in FINGER, with half receiving usual medical advice and the other half undergoing this intensive multidomain intervention, resulting in better overall cognitive performance and fewer chronic diseases (11, 13). These promising findings led to similar multidomain interventions worldwide, taking into account regional characteristics (e.g., variations in dietary habits or national guidelines for managing hypertension, diabetes, and dyslipidemia) (12, 14–16), while relevant brain health clinics have been founded worldwide (17, 18).

The purpose of this paper is to provide a detailed presentation of the Greek Interventional Geriatric initiative to Prevent Cognitive Impairment and Disability (GINGER) study protocol, which is based on the FINGER study principles. GINGER s a multidomain intervention to reduce the risk of dementia in a Greek cohort. Specifically, it focuses on individuals with subjective cognitive complaints who visit memory clinics, dementia daycare centers, geriatric psychiatry clinics, or other related facilities, and for whom the thorough examination does not reveal cognitive abnormalities and the diagnostic criteria for either mild cognitive impairment (MCI) or dementia are not met. This phenotype sets the stage for targeting subjective cognitive decline, which is linked to an increased risk of developing dementia (19, 20). In particular, compared to individuals without subjective cognitive complaints, people with such complaints have 1.4 to 2.2 higher risk for developing MCI and dementia with a shorter conversion time (21–23). In the absence of available pharmacological or other biological strategies to address their complaints, individuals who would progress to detectable cognitive decline, could benefit from personalized interventions targeting the modifiable risk factors reviewed above, while such interventions may also exert beneficial effects on individuals with subjective cognitive complaints in whom subjective cognitive decline would fully remit or remain stable at follow-up (24). Adopting a precision medicine approach, and to reduce participant burden from receiving all interventions, GINGER implements needs-based personalized interventions targeting specific risk domains: cognitive empowerment, healthy eating, regular physical activity, management of depression, anxiety and sleep disturbances, metabolic regulation, and management of sensory impairments. The findings of GINGER will form a solid basis for laying foundation for the creation of a network of second-generation memory clinics, called “Brain Health Services” in Greece (25), which are underpinned by the personalized and precision-medicine principles. Such clinics offer services for a segment of the population without cognitive impairment who wish to preserve or improve their cognitive function, and for whom there is a lack of specific programs in current memory clinics.

## Methods and analysis

### Objectives

This pilot implementation of the comprehensive, personalized, six-month, multi-level intervention aims to examine the feasibility of the protocol, based on

- the number and type of interventions which are chosen by each participant from those recommended to them;
- participant adherence by measuring the percentage of sessions of each intervention in which the beneficiary participated;
- participant satisfaction based on completing parts of a satisfaction questionnaire

Furthermore, the impact of each intervention on the specific risk domain it addresses considering cultural nuances and socio-economic factors, will be evaluated using measures specific to each intervention (e.g., changes in dietary habits, physical exercise, anxiety/depressive symptoms, sleep, regulation of metabolic parameters, etc.). Lastly, the effects of the intervention on cognitive function, dementia risk and quality of life are assessed. The findings will guide necessary refinements to the protocol, so that the intervention becomes more easily and efficiently applicable.

### Overview of study

**Figure 1:**
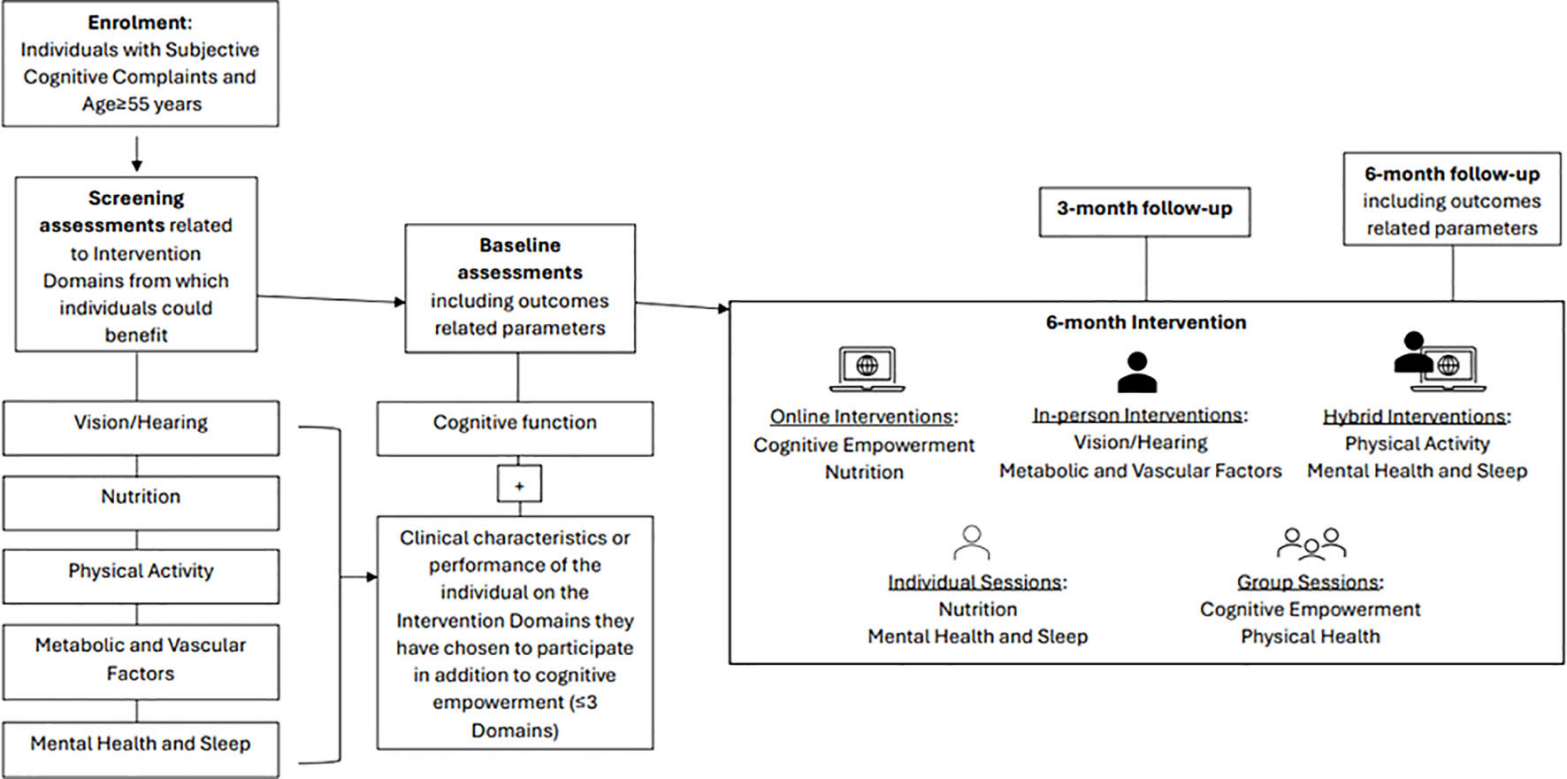
Overview of the procedures of the Greek Interventional Geriatric Initiative to Prevent Cognitive Impairment and Disability (GINGER) in individuals with subjective cognitive decline.

This GINGER pilot implementation is a proof of concept, pragmatic study. A transdisciplinary team assesses each participant and develops with him/her a personalized intervention plan targeting modifiable risk factors relevant to each individual. There are six different intervention arms, being a modified version of the FINGER intervention model (13). These focus on cognitive performance, nutrition, physical activity, vision and hearing correction, management of vascular and metabolic parameters (i.e. hypertension, dyslipidemia or diabetes), smoking cessation, alcohol consumption, as well as treatment of depression, anxiety and/or insomnia and sleep disturbances. In addition to the cognitive empowerment intervention, everyone has the opportunity to choose up to three different arms of intervention among those recommended based on the results of the selection assessments and their personal preferences. The outcomes are assessed three and six months after baseline. **Figure 1** illustrates the flowchart outlining the sequence of procedures followed in GINGER.

### GINGER network and sites

The GINGER network consists of clinical and/or research centers which bring together expertise from various fields and contribute to a comprehensive trans-disciplinary approach. The following Dementia Day Care Centers participate in the network:

- Dementia Day Care Center of Alzheimer Athens in Athens,
- Day Care Center for people with dementia of the Society of Psychosocial Research and Intervention in Ioannina,
- Alzheimer Day Care Center at the General University Hospital in Heraklion,
- Patras Dementia Day Care Center of the Corporation for Succor and Care of Elderly and Disabled-FRODIZO.

In addition, the clinicians and researchers of following university departments are members of the GINGER network:

- 1st Neurology Department of the National and Kapodistrian University of Athens,
- Departments of Psychiatry and Neurology of the University of Crete,
- Department of Nutrition and Dietetics of the Harokopio University,
- Department of Computer Science of the Ionian University,
- Departments of Psychiatry and Physiotherapy of the University of Patras.

The Department of Neurology of the University of Thessaly, the Dementia Day Care Center in Larissa and three private practices in Thessaloniki are GINGER associated members. They will become active members once the pilot is completed.

The intervention arms are delivered hybridly, online or in- person. On site assessments and interventions are delivered at dementia day care centers in Athens, Heraklion and Ioannina, at University Hospital-based outpatient clinics in Heraklion and Patras and at the Laboratory of Clinical Physiotherapy and Research (CPR lab) of the Department of Physiotherapy in Patras.

### Participants

The pilot implementation of GINGER includes 50 individuals aged 55 years and above diagnosed with subjective cognitive decline during the screening phase. Inclusion criteria are (a) subjective cognitive complaints confirmed by the SCD Questionnaire score part I, MyCog (SCD-Q >7) (26), (b) absence of objective cognitive decline, as verified by normal performance on the Montreal Cognitive Assessment (MoCA) based on normative data for the Greek population (27).

Exclusion criteria are (a) diagnosis of MCI or dementia of any cause based on international diagnostic criteria (e.g. (28–30), (b) presence of chronic mental or neurological disorders or unstable physical illnesses affecting cognitive function (e.g., schizophrenia, bipolar disorder, chronic depression (31), multiple sclerosis, history of traumatic brain injury, hydrocephalus, Parkinson’s disease, epilepsy, thyroid disorders), (c) age <55 years, (d) poor command of Greek, (e) no access to smart phone or other modern technology equipment and/or low familiarity with applications related to teleconferencing, (f) severe sensory impairments hindering verbal communication.

Individuals meeting entry criteria undergo a series of brief assessments to quantify which risk factor domains (physical activity, depression, nutrition, etc.) apply to them thus suggesting additional interventions, beyond cognitive empowerment, from which they may benefit towards reducing dementia risk (baseline assessment). These screening assessments, which are administered by GINGER staff to people with subjective cognitive complaints, are summarized in **Table 1**. After a thorough description of the purposes and the procedures of each intervention, from which each beneficiary can benefit according to the findings of the series of brief assessments, beneficiaries are asked to decide on which additional interventions they would like to participate in. The upper limit of additional interventions was arbitrarily set at three being a reasonable compromise between the five available in GINGER additional interventions for managing modifiable risk factors of dementia in mid- and late life and a pragmatic, intensive six-month intervention program, which aspires to be beneficiary-friendly and can be successfully completed.

**Table 1:** Screening and detection of intervention domains from which the beneficiaries can benefit.

Domain	Method	Cut-off
Cognitive function screening		
	SCD-Q	>7
	MoCA	education ≤6 y/o: >23 education >6 y/o: >26
Beneficial Interventions Alongside Cognitive Empowerment		
Hearing and Vision		
	MA1, MAICO Diagnostics	45-55 y/o 1000Hz: 30dB- 4000Hz: 40dB 55-65 y/o 1000Hz: 30dB- 4000Hz: 40dB >65 y/o 1000Hz: 35dB- 4000Hz: 45dB
	Peek Acuity Pro, Peek Vision	PEEK Acuity Binocular Score = [0.2 to 1] LogMAR (≤ 6/9.5 and >6/60 in Snellen meters)
Dietary Screening		
	MedDietScore	≤2 in at least 3 food groups (olive oil and alcohol are excluded)
Physical Activity		
	IPAQ	600 MET
	2min Walking Test	Available normative values
	Hand Grip Test	Available normative values
Depression, Anxiety and Insomnia		
	HADS	≥8
	AIS	≥6
	STOP-BANG	≥3
Metabolic and Vascular Risk Factors		
	BP	Systolic BP >130mmHg and/or diastolic BP >85mmHg
	TGL	≥150mg/dl
	HDL	Men <40mg/dl, Women <50mg/dl
	LDL	≥130mg/dl
	Glucose	FPG >100mg/dl and/or HbA1c >6%
	Smoking	Any current smoking
	Heavy Drinking Alcohol	Men: >4 drinks/day or >14 drinks/week; Women: >3 drinks/day or >7 drinks/week
	History of Stroke and/or MI	Physician diagnosis

SCD-Q, Subjective Cognitive Decline Questionnaire; MoCA, Montreal Cognitive Assessment; IPAQ, International Physical Activity Questionnaire; PSSQ, Penn State Sleep Questionnaire; AIS, Athens Insomnia Scale; HADS, Hospital Anxiety and Depression Scale; BP, Blood Pressure; TGL, Triglycerides; HDL, High-Density Lipoprotein; LDL, Low-Density Lipoprotein; FPG, Fasting Plasma Glucose; HbA1c, Glycosylated Hemoglobin; MI, Myocardial Infarction.

Before the start of interventions the following data are recorded for all beneficiaries of the intervention: demographic and anthropometric data (height, weight, waist, hip, and body mass index), medications with calculation of the anticholinergic burden scale (32), Cardiovascular Risk Factors, Ageing and Dementia (CAIDE) score (33) and Lifestyle for Brain Health (LIBRA) index (34). In addition, baseline assessment includes quality of life and daily functioning, which is assessed with the Short Form Survey Instrument SF-12, administered at the 3- and 6- month follow-ups (35). The 3- and 6-month follow-up also includes completion of the Greek version of the Client Satisfaction Questionnaire-8 for the measurement of outpatient satisfaction which is related to the behavior of medical and non-medical healthcare professionals (36). The 3- and 6-month follow-up also include the MoCA and the SCD Questionnaire score part I, MyCog.

### GINGER interventions and assessment of their effects

#### Cognitive empowerment

The following assessment tools for cognitive function are administered prior to the start of the intervention: Greek Verbal Learning Test (GVLT), Trail Making Tests (TMT) A & B, Color-Word Interference (Stroop) Test, Digit Span Test (included in the fourth revision of the Wechsler Adult Intelligence Scale- WAIS), Rey Complex Figure Test (RCFT), Digit Symbol Substitution Test (DSST) (included in the Wechsler Adult Intelligence Scale–Revised (WAIS-R), Boston Naming Test-short form (15-item), F-A-S Phonemic (FAS) and Semantic Verbal Fluency Tests (37–43). The outcomes of the intervention are performance on GVLT, Trail A & B, Stroop, Digit Span WAIS IV, RCFT, DSST WAIS-R, Boston Naming Test-short form (15-item), and Phonemic and Semantic Fluency Tests, which will be administered at the six-month follow-up assessment.

The intervention is conducted online by certified, experienced neuropsychologists. Based on mounting evidence (44), the intervention consists of two group sessions per week, each lasting 60 minutes. Each group consists of 5-6 individuals, and the entire intervention takes place online using platforms such as Zoom/Skype/Viber. Each individual should attend at least the 70% of the total cognitive empowerment sessions delivered by the program. Otherwise, he/she is not included in the final number of participants. The session content encompasses memory and attention exercises alternating with a weekly program of language performance improvement and executive function exercises, such as programming, cognitive control, reasoning/critical thinking exercises. The memory and attention empowerment program includes eight exercises based on written text (questions about the text content, information recall exercises, attention exercises - concentration and selective attention). The language program consists of eight exercises (naming, finding synonyms, verbal and semantic fluency, word finding, sentence order finding, comprehension exercises, and written performance). After 20 minutes in each session, participants are asked to provide their answers, and the therapist discusses correct answers with the group. The executive functions improvement program includes eight exercises (quizzes, puzzles, problem-solving, reasoning exercises, cognitive rotation exercises, Raven matrices, critical thinking exercises, basic mathematics, and letter sequence). Participants receive specific instructions and are given 45 minutes to complete the exercises. Subsequently, for 15 minutes, all participants together with the therapist discuss the answers, and correct answers will be provided. The structure of each session (e.g., if the facilitator conducts eight exercises or fewer) is determined based on the performance of the participants. The therapist team conducting the sessions records attendance and the progress of each participant in a separate Google Drive account in an Excel sheet with pseudonyms based on individual comments/notes and the therapist’s subjective assessment.

#### Nutritional intervention

Participants are screened for their adherence to the Mediterranean Diet, calculated using the MedDietScore (45). Additional assessments for those included in the nutritional intervention include an evaluation of dietary intake using the 24-hour recall methodology. Specifically, participants are asked to recall their dietary intake from the previous day, repeated for 3 days (2 weekdays and 1 weekend day) over a period of 10 days. The 24-hour recalls are conducted via telephone calls (only the first could be done in person), following a standardized methodology for recall and recording (46). The collected information is analyzed for energy and nutrient intake, food consumption, and food group consumption. The outcomes of the intervention are the MedDietScore and the consumption of individual food groups. People included in the nutritional intervention are re-assessed regarding adherence to the Mediterranean Diet at the end of the 3rd and the 6th month of the intervention.

The intervention aims to enable participants to make necessary dietary changes to adhere as much as possible to a Mediterranean diet, by increasing the frequency of consuming foods that characterize the Mediterranean dietary pattern (47). The nutritional counseling sessions are conducted online by an experienced dietitian who has received relevant training from the research team members.

The intervention consists of seven individual, online nutritional counseling sessions, each lasting 40 minutes, conducted every two weeks for the first two months and then monthly for the rest of the intervention period. The nutritional counseling is based on goal-setting theory (48). Additionally, motivation and incentive strategies are utilized, such as exploring readiness, self-monitoring, stimulus control and problem-solving techniques, managing high-risk situations, relapse prevention training, and positive feedback. A visual agenda-setting diagram is developed (either on paper or electronically) and used for goal setting and evaluation. Beneficiaries are encouraged to identify their priority goals and propose possible changes to their diet to achieve and maintain their respective goals. Each session also includes an educational component to assist in goal achievement (informational discussions and familiarization with foods that constitute the Mediterranean diet, seasonal shopping lists, meal plans, and/or diets). Portions are determined based on each individual’s energy needs, with no emphasis on weight loss, although potential changes in weight will be monitored and recorded.

#### Physical exercise intervention

Screening for inclusion in Physical Exercise Intervention is based on the results of instruments assessing physical activity, such as the International Physical Activity Questionnaire (IPAQ-7), the 2min walk test and the Hand grip test (49, 50). Participation in the intervention is preceded by an assessment of cardiovascular health by a cardiologist, certifying that the individual’s cardiovascular health state allows him/her to participate in physical exercise activities. Prior the start of the intervention, the Sit to stand test, miniBESTest and Falls Efficacy Scale International (FES-I) are administered and enable the development of an individualized intervention program, progressively adjusting the difficulty level (51–54). The effectiveness of the intervention is evaluated at the end of the 3^rd^ month and at the end of the 6^th^ month of the intervention using the 2min walk test, the Hand grip strength test, the Sit to stand test, the miniBESTest and the FES-I, administered randomly to each individual.

The physical exercise intervention consists of three sessions per week, one conducted in person with a small group of up to 5 individuals under supervision, one through tele-exercise, and one at home without supervision. Experienced physical therapists and trainers, adequately trained, assess and conduct exercise sessions at each GINGER center and are supervised by staff of the Department of Physiotherapy of the University of Patras.

A typical in-person or online exercise session is structured as follows: five minutes warm-up, five minutes cool-down, and 40-50 minutes of exercise, including 20 minutes of aerobic exercise (AE), 20-30 minutes of resistance exercise (RE), and balance exercise (BE). The AE has moderate to high intensity, 60%-85% of the Targeted Heart Rate (THR) calculated using the Karnoven formula and the Borg RPE scale (55). For RE and BE exercises, there is a cyclic intervention program with breaks (one minute rest between sets), with each exercise performed in two sets targeting major muscle groups. RE exercises consist of eight to twelve repetitions at an intensity of 50%-80% of the ten Repetition Maximum (10RM). Each BE exercise involves up to ten repetitions with holds of 5”-10”. All exercises are evaluated every three to four weeks to monitor progress/improvement. Progress is assessed based on the Borg RPE scale (6/10) and 10RM for RE. For the home program, instructions are given for performing AE exercises (walking) in THR for a total of 30 minutes, individually, once per week. Distance, heart rate, walking pace, etc., are recorded using a smartwatch. The assessment of exercise program adherence is based on weekly exercise logs and monthly completion of the Exercise Adherence Rating Scale (EARS) (56). Efforts to improve compliance will include weekly reminders via SMS or phone calls, communication with the head physical therapist in case of two consecutive absences, and regular feedback from the therapist regarding individual progress and achievements in relation to their physical condition.

#### Hearing and vision correction intervention

GINGER beneficiaries in whom the screening assessments of auditory function using portable audiometer (MA 1, MAICO Diagnostics) and of visual acuity with the Android smartphone application Peak Acuity Pro (Peek Vision Ltd) reveal hearing impairment in at least one ear and/or visual difficulties in at least one eye, respectively, are referred to an otolaryngologist and/or ophthalmologist of their choice for further investigation of sensory function and correction. At baseline, participants with detected hearing- and/or vision deficits complete the Hearing Handicap Inventory for Adults-Screening version (HHIE-S) (57) and the Veterans Affairs Low-Vision Visual Functioning Questionnaire (VA LV VFQ-48) (58), respectively. At the end of the 3^rd^- and the 6^th^ month of the intervention, compliance with the recommendations of the otolaryngologist and/or ophthalmologist (use of hearing aids, corrective lenses) is evaluated with relevant items of the Brief Adherence Rating Scale (BARS) (59), the HHIE-S and VA LV VFQ-48 questionnaires are completed, and the above-mentioned hearing and vision assessment tests are repeated.

#### Intervention for vascular and metabolic parameters control

If the screening process of vascular and metabolic parameters indicates the need for intervention in areas such as blood pressure, LDL cholesterol-, glycated hemoglobin- and fasting blood sugar levels, smoking habits, increased alcohol consumption, and/or the participants have suffered a stroke and/or a myocardial infarction, they are referred to internists or cardiologists of their choice. The aim of this referral is the regulation of the metabolic-vascular parameters requiring pharmacological-behavioral intervention, the reduction of alcohol consumption, and the decrease or cessation of smoking. At the end of the 3^rd^ and 6^th^ month of the intervention the blood pressure readings, findings of the biochemical blood parameter re-measurements, and the use of tobacco and alcohol consumption are recorded. Additionally, any cerebrovascular event and/or myocardial infarction are documented. The laboratory tests are performed using similar analytical methods.

#### Intervention for depression, anxiety, and insomnia

Individuals participating in GINGER are screened for depression and anxiety using the Greek version of the Hospital Anxiety Depression Scale (HADS) (60) and for insomnia/sleep apnea syndrome using the Athens Insomnia Scale (61) and the STOP-BANG scale (62), respectively. If HADS score ≥8, indicating depressive/anxiety symptoms, AIS score ≥6, indicating insomnia, and/or STOP-BANG score ≥3, indicating sleep apnea, they are further evaluated for the symptom group(s) they screened positive (depression, anxiety and/or insomnia). Instruments that are administered through the electronic platform include State-Trait Anxiety Inventory form Y (STAI Y), Perceived Stress Scale - 14 items, PennState Sleep Questionnaire, and Fatigue Assessment Scale (FAS) (63–66). Participants are also clinically assessed in person for the presence/severity of depression/anxiety symptoms by experienced psychiatrists specialized in geriatric psychiatry. Sleep is evaluated through telepsychiatry by an experienced sleep medicine-certified psychiatrist. The assessment of the effectiveness of the intervention is conducted three months after the enrollment of each beneficiary and at the end of the intervention using the HADS, Perceived Stress Scale - 14 items, AIS, and FAS tools.

**Figure 2:**
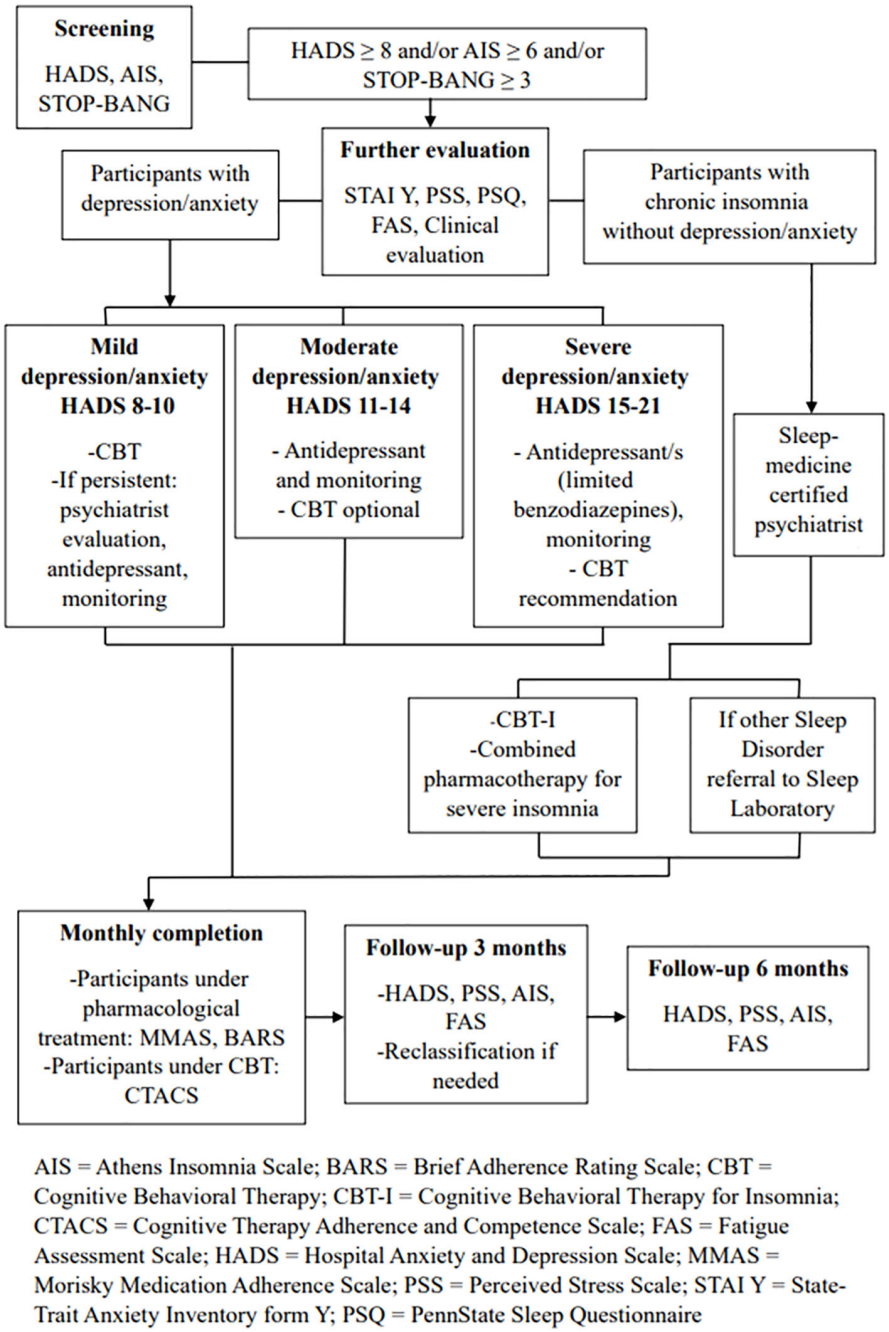
Overview of the intervention for treating anxiety, depression and/or insomnia.

Regarding depression/anxiety symptoms, based on the results of the HADS, participants are classified into three groups according to symptom severity (67): mild depression and/or mild anxiety group (8-10 score in the respective subscales of depression or anxiety in HADS), moderate depression and/or moderate anxiety group (score in the corresponding HADS subscales 11-14), or severe depression and/or severe anxiety group (score in the corresponding HADS subscales 15-21). This is necessary, as the intervention differs by symptom severity. Participants without depression or anxiety who report symptoms of insomnia/poor sleep are further evaluated for fulfilling the criteria of the International Classification of Sleep Disorders-third edition (ICSD-3) (68). The intervention is illustrated in **Figure 2**.

Individuals with mild depression and/or mild anxiety undergo internet-based cognitive-behavioral therapy (CBT) consisting of 8-10 weekly sessions. The sessions are individual and online in all cases, are conducted by a small team of five accredited CBT experienced therapists (psychologists, psychiatrists). The team is supervised and coordinated by an accredited CBT supervisor, coordinator of the 4-year CBT educational program offered by the Division of Psychiatry and Behavioral Sciences, School of Medicine, University of Crete, Greece. The structure of each CBT session follows a specific protocol. Clinical reevaluation is conducted at the end of this CBT intervention.

If symptoms persist, pharmacotherapy with an antidepressant (e.g., citalopram, escitalopram, sertraline, mirtazapine, trazodone) is initiated by a psychiatrist at the center where the individual was recruited and examined. After the initiation of pharmacotherapy, reassessments take place every four weeks, and necessary adjustments are made. If symptoms improve, individuals are clinically reassessed either three months after the visit where symptom relief was observed, or at the end of the intervention period. If symptoms persist, individuals are reevaluated once a month until the end of the six-month period.

Participants with moderate depression and/or moderate anxiety receive treatment with an antidepressant and they also have the option to participate in CBT intervention. Both treatments and follow-up are conducted as described previously.

Participants with severe depression and/or severe anxiety, according to the results on the corresponding HADS subscales, receive treatment with one or more antidepressants in this case, while the combination of pharmacotherapy with a 10-session CBT intervention is recommended. Participants are clinically reassessed every four weeks until symptom relief, and then every two months after symptom relief, as well as at the end of the study. The use of benzodiazepines or similar medications is reserved for critical cases, where they are absolutely necessary (e.g. active suicidal ideation).

If there is an exacerbation of depression and/or anxiety symptoms at the 3-month follow-up based on the HADS results, reclassification of participants into the appropriate intervention group takes place, and the corresponding treatment is initiated according to the aforementioned schemata.

Participants with chronic insomnia without depression/anxiety undergo individual, online Cognitive Behavioral Therapy for Insomnia (CBT-I) consisting of eight sessions based on the J. Edinger(2008) protocol (69). Additionally, among those reporting severe insomnia symptoms (total sleep time less than 5 hours/24 hours and/or severe daytime symptoms related to functional impairment), a pharmacological intervention is conducted, combined with CBT-I (**Figure 2**). Participants receive mirtazapine 7.5-15 mg every night. They are initially reassessed by a psychiatrist, sleep specialist, or physician specializing in sleep medicine after four weeks. If the pharmacological treatment is effective in subjective sleep improvement and has no significant side effects, participants are clinically reassessed after five months (at the end of the intervention). If, during the first reevaluation, the pharmacological treatment for chronic insomnia proves ineffective or has significant side effects, it may be modified, and participants are reassessed after 4 weeks. This process can be repeated as needed.

Participants with other sleep disorders as indicated by history, such as sleep apnea, for the detection of which the STOP-Bang screening tool is used (scores > 2 indicate the presence of sleep apnea), or other disorders (restless legs, parasomnias, etc., based on the PennState Sleep Questionnaire), are referred to sleep experts in their area and the necessary diagnostic and therapeutic measures are initiated.

The assessment of adherence to pharmacological treatment for the participants under pharmacological treatment is based on monthly completion of the 8-item Morisky Medication Adherence Scale (MMAS) and the BARS (70). Adherence to CBT intervention is evaluated using the 21-item Cognitive Therapy Adherence and Competence Scale (CTACS) for depression and anxiety interventions (71). Additionally, after each CBT session, a checklist for agenda adherence developed for this specific protocol is completed by the therapist. To ensure treatment fidelity and administration according to the protocol, the coordinator provides supervision to all therapists through bi-monthly or monthly video conferences throughout the intervention. To ensure that therapists follow the prescribed therapy guidelines and properly document that the treatment is provided in a standardized way, all CBT sessions are recorded, and a random portion (10%) is scored with a content checklist by an independent rater (accredited CBT therapist-supervisor). If fidelity checks on the therapeutic protocol reveal that a therapist deviates from the protocol, the independent rater provides corrective feedback to minimize this deviation over time.

#### Data management

**Figure 3:**
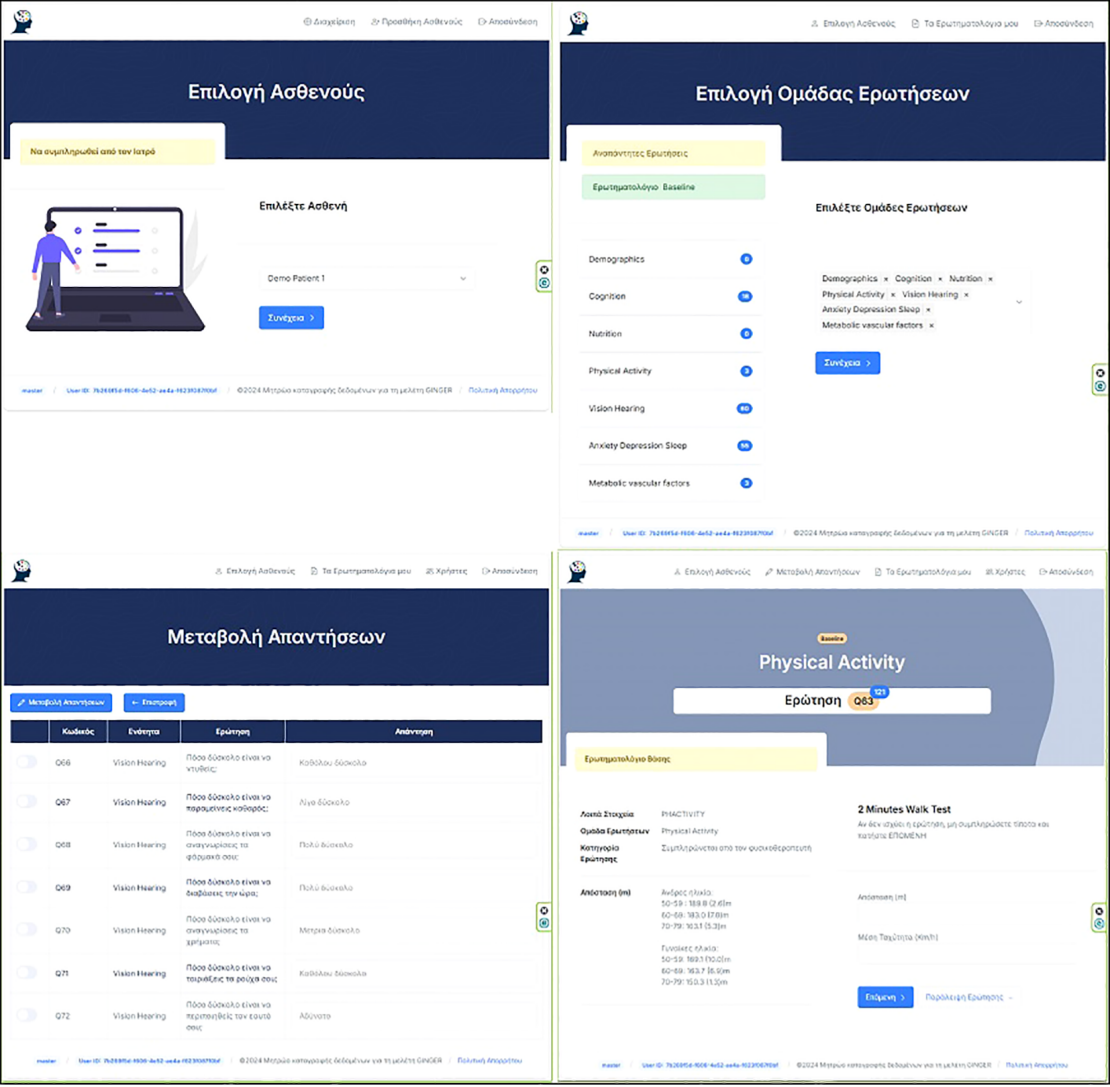
**<**Online data collection platform – main screens.

**Figure 4:**
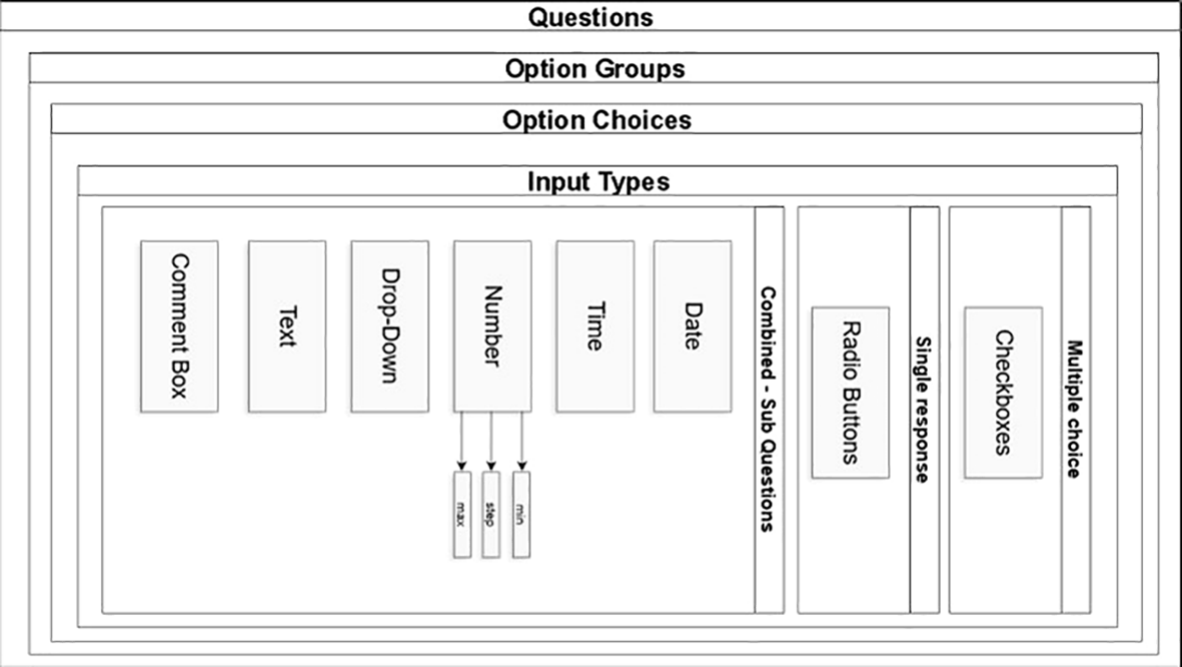
Question in a form of container.

Data collection is paper-based (e.g. cognitive assessment data) and web-based (demographic and clinical information, questionnaire responses, and records of online sessions). Data are directly or in time proximity to their collection in paper- format entered in a pseudonymized form in a secure online digital database created and hosted by the Department of Informatics at the Ionian University (**Figure 3**). The protection and secure storage of this material is ensured using appropriate methods that meet the necessary requirements established by the General Data Protection Regulation (GDPR - 2016/679) of the European Union. The online questionnaire is a secure web-based application developed for clinical research and designed to support the composite form of questions and sub-questions required in GINGER. The question module is designed in the form of container that is assigned to a group of options providing this way the potential for option groups to be assigned to many questions expediting the rewrite of complex questions in cases that they share the same group of options. (e.g., questions in cognition with GVLT group of options) **Figure 4**. From the technology perspective, the application was developed on CakePHP MVC framework, with the Bootstrap component library for responsive behavior. Questionnaire answers and survey templates are exported in either PDF or CVS format for further statistical processing. The backend relational database (RDBMS) is designed in a way that it can be deployed to any database engine such as Microsoft SQL, Oracle or MySQL. The software was developed by implementing the latest security aspects of Application Security Lifecycle guidelines and the Shift-left Secure Coding principles. During application deployment, penetration testing was performed by the Information Security team of Ionian University with medium and low severity findings that remediated. The authentication and authorization modules were designed to facilitate the domain expert who is authenticated in the system to fill out certain section(s) in survey on behalf of a participant, if authorized to do so. Thus, we implemented a one-to-many design between participants and domain experts allowing a participant to encounter many domain experts according to the number of sections in a questionnaire. Consequently, a domain expert can fill out sections in questionnaire but only for participants who he/she is authorized to. The level of system moderators is authorized to update and modify authorizations for participants and domain experts. From built-in security perspective, the GINGER Survey software supports state of the art security policies and modules such as Content Security Policy, Security Headers, Cross-Site Request Forgeries (CSRF) protection, Form tampering prevention, bcrypt Password Hashing, ModSecurity (WAF) application firewall module, HTTPS enforcement and GeoIP blocking allowing connections (access) only from certain countries. Finally, data encryption at rest (DARE) is implemented in database storage layer to ensure that the data is securely stored encrypted in database. Decryption keys are stored in GINGER software ensuring that only the software can access survey data and thus if someone gains access directly to database, data will remain encrypted.

#### Ethical considerations

Participation in the intervention is voluntary and is preceded by a thorough description of its purposes and procedures. Written informed consent is obtained from all GINGER participants. GINGER fully complies with the principles of the Helsinki Declaration and its revisions. It has been approved by the University of Patras Bioethics Committee (15244), and the Patras General University Hospital Bioethics Committee (256/13.09.2022) and subsequently by the Scientific and Bioethics Committees of all participating institutions.

No invasive procedures beyond blood collection for the identification and control of vascular and metabolic risk factors and sample collection of plasma and serum take place. Any potential rare side effects, such as pain, bleeding or swelling are explicitly disclosed, with an emphasis on their temporary nature. Participants do not incur any financial burden for participation in GINGER. Of note, at baseline, participants undergo a thorough cardiovascular examination before engaging in physical exercise interventions, ensuring that participation in such activities does not pose any health risk for them.

### Intervention design innovative aspects

GINGER is characterized by several innovative features which are expected to ensure its feasibility and efficacy. Firstly, GINGER is not structured as “one-size-fits-all” but rather as a precision prevention approach (72). Through an extensive baseline assessment, GINGER identifies the intervention domains from which the participant is most likely to benefit. This strategy of tailoring interventions to individual needs and preferences aims to safeguard effectiveness and time-efficiency as well as reduce participation burden therefore enhancing adherence. Secondly, the mental health intervention is not restricted to the management of depressive symptoms. It considers anxiety and sleep symptoms as well as depression, which, if present, are treated with a structured online CBT intervention and/or pharmacotherapy. Moreover, hearing and vision assessment and support add valuable arrows to the prevention quiver (73). The nutrition arm promotes the Mediterranean diet, known for its beneficial effects on dementia and cognitive decline, outperforming other nutritional interventions (74). In addition, new technologies are used for screening and follow-up assessments, (self-) monitoring (e.g. daily calendars, Exercise Adherence Rating Scale) and feedback from health professionals (rewarding, support) and peer support (chat). Another novel aspect of the intervention is its two-axes hybrid character: delivery through both individual and group sessions, as well as online and in-person. Last but not least, this initiative embodies a first step towards mapping the frequency of the presence of dementia risk factors in people with SCD in different regions of Greece.

### Anticipated results

The pilot phase of GINGER aims to investigate the feasibility of the intervention protocol, so that necessary refinements can be made allowing the intervention to become more easily and efficiently applicable. The relationship between the number and the types of interventions in which the beneficiary was involved and respective adherence will inform necessary refinements in the upper limit of the number of total interventions and feasible combinations of interventions in which a person can participate. It will help to reach a reasonable compromise between maximizing the number intervention arms in which each participant is involved while maximizing compliance. Moreover, the intervention takes place at different settings, i.e. community-based day care centers, university hospital-based outpatient clinics, university-based laboratories. Brain health services are provided in variable settings so far (72). Findings of the analyses may shed light on the most appropriate setting for such services in Greece (e.g. community-based dementia day care centers *vs*. university hospital-based outpatient clinics). Finally, since no similar previous studies have been conducted in Greece, the project will provide valuable information about whether and to what extent models being implemented in other countries with different cultural, and socioeconomic backgrounds can be adapted in this Southeastern European country.

Data analyses will initially involve estimating means and confidence intervals for continuous variables and rates for categorical variables. Additionally, individual level pre- to post- comparisons to assess the magnitude of change that might be anticipated will be assessed using both univariate and multivariate statistical models. The main outcome measures will include (i) number and types of recommended interventions in which participants chose to participate, (ii) percentage of participants who did not adhere at all, partially complied, or adequately adhered in the first three months and throughout the study, (iii) participant satisfaction degree and (iv) changes over the intervention period in the domains targeted by the interventions in which the beneficiary participates, in cognitive performance, the CAIDE- and LIBRA score and quality of life, between baseline and the 3- and 6-month follow-ups. Compliance lower than 50% is considered partially adequate and is scored with one point. Compliance over 50% is considered adequate and is scored with two points, while non-adherence with the interventions is scored with zero in line with previous reports (75). These scores are summed and divided by the sum of scores the individual would receive if he/she was adequately compliant with all interventions he/she chose to participate at baseline, so that the overall compliance with the intervention is derived. Associations between the outcome parameters of interventions at baseline, follow-ups and over time will also be studied (e.g., associations between cognitive performance and depressive symptoms) by computing univariate and multivariate models. Furthermore, relationships with other parameters, such as changes in quality of life, adherence to therapist advice etc., will be explored. Finally, regression models will be utilized to identify additional factors (e.g., demographic factors, the number of interventions in which the beneficiary participated) associated with the key outcome measures.

The following pre-defined criteria will be used to inform the decision about whether and to what extent changes are necessary before upscaling the implementation of the GINGER protocol (76). It will be deemed appropriate to progress without refinements, if the overall compliance with the intervention is ≥0.7, drop-out rate is ≤0.2 and the score on the 4-point Likert scales of the responses to the Client Satisfaction Questionnaire- is ≥3.5. The “Stop” criteria will consider if the overall compliance is ≤0.3, the drop-out rate is ≥0.4 and the mean score on the 4-point Likert scales of the responses to the Client Satisfaction Questionnaire- is ≤1.4. In this case, the protocol will undergo fundamental changes. If the assessment outcome falls between the “Go” and “Stop” criteria the necessary refinements will take place based on selected data.

### Pitfalls, limitations and troubleshooting

The pilot phase of GINGER has various pitfalls and limitations. Participant drop-out and low adherence are crucial pitfalls that need to be addressed. To minimize drop-out and increase adherence, the assessments are conducted, and the interventions are delivered in most cases, as combinations of online and in-person sessions. Regular communication with beneficiaries is maintained via in-person and online meetings, social media and face-to-face meetings with the local therapeutic teams. Risk of drop out and/or low adherence may be reduced through the relative short duration of the intervention, since earlier dementia prevention interventions lasted one year or longer (72). In addition, tailored approaches, like that of GINGER, which follow the principles of personalized medicine (77), seem to ensure adherence, since they meet the individual needs of beneficiaries who are involved only in interventions from which they can benefit and not in additional intensive interventions which do not meet their individual needs regarding the management of dementia risk factors (72).

The multicentric and longitudinal nature of the intervention design may lead to variability in data collection because of instructor bias. The risk is minimized through detailed protocols, extensive training, partial direct online data collection and constant supervision of the therapeutic teams by the heads of each intervention arm.

The assessment of the effects of the GINGER relies on within person longitudinal changes of participants and is not compared to standard care, placebo, general information/health advice, or sham, while the sample size is relatively small. Since there is robust evidence regarding the effectiveness of cognitive decline prevention programs (10), GINGER has not been designed as a parallel group clinical trial. It is a pragmatic cognitive decline prevention program paving the way towards brain health clinics in Greece.

## Conclusion

Real world interventions offer to cognitively unimpaired individuals the opportunity to act and reduce their risks for cognitive decline and developing dementia in the future. GINGER is a 6-month pragmatic, precision-medicine based multidomain intervention which is structured along both in-person and online sessions. Its pilot study assesses the feasibility of the protocol, so that the necessary refinements are implemented, and the protocol can serve as the backbone of future brain health clinics in Greece.
